# Clinical evaluation of patients with a neuropsychiatric risk copy number variant

**DOI:** 10.1016/j.gde.2020.12.012

**Published:** 2021-06

**Authors:** Samuel JRA Chawner, Cameron J Watson, Michael J Owen

**Affiliations:** 1 MRC Centre for Neuropsychiatric Genetics and Genomics, School of Medicine, Cardiff University, UK; 2 Cardiff University Centre for Human Developmental Science, School of Psychology, Cardiff, UK; 3 Preventive Neurology Unit, Wolfson Institute of Preventive Medicine, Queen Mary University of London, UK; 4 Barts Health NHS Trust, London, UK

## Abstract

Several copy number variants (CNVs) have been identified to confer high risk for a range of neuropsychiatric conditions. Because of advances in genetic testing within clinical settings, patients are increasingly receiving diagnoses of copy number variant genomic disorders. However, clinical guidelines surrounding assessment and management are limited. This review synthesises recent research and makes preliminary recommendations regarding the clinical evaluation of patients with neuropsychiatric risk CNVs. We recommend multi-system assessment beyond the initial referral reason, recognition of the potential need for co-ordinated multidisciplinary care, and that interventions take account of relevant multimorbidity. The frequently complex needs of patients with CNVs across the life-course pose challenges for many health care systems and may be best provided for by the establishment of specialist clinics.

**Current Opinion in Genetics and Development** 2021, **68**:26–34

This review comes from a themed issue on **Molecular and genetic basis of disease**

Edited by **Jennifer Gladys Mulle**, **Patrick F Sullivan** and **Jens Hjerling-Leffler**

For a complete overview see the Issue and the Editorial

Available online 15th January 2021

**https://doi.org/10.1016/j.gde.2020.12.012**

0959-437X/Crown Copyright © 2021 Published by Elsevier Ltd. This is an open access article under the CC BY license (http://creativecommons.org/licenses/by/4.0/).

## Introduction

Several copy number variants (structural deletions, duplications or translocations, >1000 bp) [1] have been robustly associated with neurodevelopmental and neuropsychiatric outcomes [2]. One of the first reports of an association between CNVs and psychiatric risk, concerned the high prevalence of psychotic disorders (30%) in adults with 22q11.2 deletion syndrome (22q11.2DS) ascertained through medical genetic clinics [3]. Advances in genomic techniques have revealed a range of pathogenic CNVs that confer psychiatric risk, often via genome-wide association studies (GWAS) that compare the frequency of CNVs in neuropsychiatric patient cohorts and controls. Through this approach risk CNVs have been identified for intellectual disability [4], ADHD [5], autism [6], schizophrenia [7,8], depression [9^•^] and bipolar disorder [10].

Medical Genetics services exist within many healthcare systems, offering both patient testing and counselling; CNVs are increasingly being detected in clinical settings through the use of technologies such as chromosomal microarray and exome/whole genome sequencing. Patient referrals can be received from a range of medical specialities and are triggered when a clinician suspects a genetic aetiology. In the case of neuropsychiatric risk CNVs, referral reasons often include, intellectual disability, developmental delay and childhood psychiatric and behavioural problems, but referrals are often from beyond psychiatry and cut across many specialities, including cardiology, neurology, paediatrics, endocrinology and speech and language services [11, 12, 13]. The process of receiving a genetic diagnosis can take several years, with some families describing long and distressing ‘diagnostic odysseys’ before they learn what is causing their child’s health problems [14]. A clear solution to this would be to implement genetic testing more widely (Ledbetter *et al*. this issue). Neuropsychiatric CNVs have been implicated in the aetiology of congenital heart abnormalities [15,16], facial dysmorphology [17, 18, 19], endocrine abnormalities [20,21], epilepsy [22,23], and anthropometric variability [24]. This highlights the pleiotropic effects of CNVs and the heterogeneity in individual outcomes.

Although neuropsychiatric CNVs as a group are relatively common, they are individually rare and almost all can be considered to be ‘orphan diseases’ (i.e. diseases affecting less than 200 000 people in the United States, equivalent to a lifetime prevalence <0.06%) [25]. Although there is increasing academic and clinical interest in CNVs generally, and the scope of research is expanding, helped in part by big data population cohort approaches to identifying outcomes [26^••^], some have been studied more intensively than others. In particular, the literature on 22q11.2DS is relatively large compared to many other CNVs, as it was already known to clinicians as syndromes such as DiGeorge syndrome [27] or Velo-cardio-facial syndrome [28] decades before current genomic testing technologies were available. Most of the focus in the literature to date has been on characterising clinical, behavioural and cognitive outcomes of neuropsychiatric CNVs, whereas research on therapeutic choices for these ‘orphan’ diseases is currently limited [25].

## Clinical evaluation

### Multimorbidity in CNV carriers frequently requires multidisciplinary care

Neuropsychiatric CNVs have pleiotropic manifestations across physical, psychiatric and cognitive domains [29^••^,^30^,^31^], and therefore carriers often have a history of disparate care from different medical specialties without a unifying diagnosis. Importantly, substantial evidence is now emerging that phenotypic changes across several CNVs follow a prescribed time-course, allowing interventional opportunity for clinical management [29^••^,^32^,^33^]. Whilst patients with neuropsychiatric CNVs can initially present to many different specialities, once a CNV has been confirmed, there is a need to consider appropriate multi-disciplinary care including access to neuropsychiatric expertise as well as the need for holistic care from relevant practitioners such as physical and occupational therapists, genetic counsellors, dieticians and social workers.

**Figure 1:**
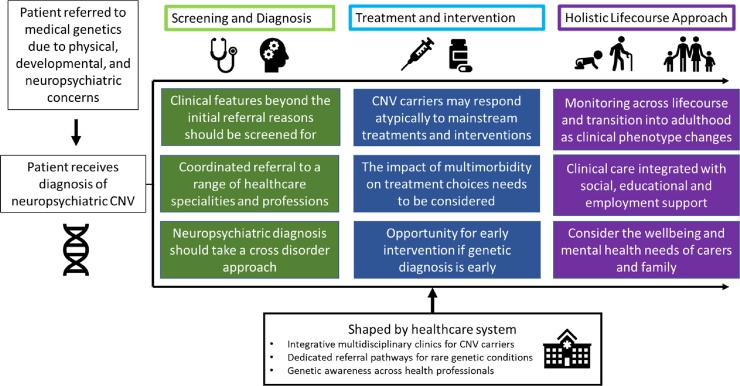
Ideal clinical examination.

We make the following broad recommendations for clinical evaluation following a diagnosis of a neuropsychiatric CNV (see Figure 1). (1) Screening broadly for medical signs and symptoms beyond the initial referral reason, as neuropsychiatric CNVs have complex pleiotropic effects (Table 1 briefly summarises and links to further literature); (2) Treatment plans should take account of the fact that CNV carriers can have atypical reactions to common treatments and that multimorbidity may affect treatment effectiveness (hence the importance of recommendation 1); (3) Neuropsychiatric CNVs have differential effects across the life-course and hence clinical management should be age-specific.

**Table 1:** Phenotypes of neuropsychiatric CNVs. These CNVs were selected on the basis of being robustly associated with neuropsychiatric conditions, and for being frequently diagnosed in medical genetic settings [29^••^,^55^]. It should be highlighted that the phenotypes listed below are not exhaustive, but highlight important features and link to useful references for clinicians and researchers

Locus	Copy number change	Neuropsychiatric	Physical health	References
1q21.1	Deletion	ADHD	CHD	[69, 70, 71, 72]
		ASD	Microcephaly	
		Epilepsy	Strabismus	
		ID	Facial dysmorphia	
		Mood Disorders		
		SCZ		
1q21.1	Duplication	ADHD	CHD (inc ToF)	[69, 70, 71, 72, 73]
		ASD	Macrocephaly	
		GAD	Facial dysmorphism	
		ID		
		Mood Disorders		
		SCZ		
2p16.3 (*NRXN1*)	Deletion	ASD	CHD	[74, 75, 76]
		ADHD	Craniofacial abnormalities	
		BPD	Seizures	
		Dysexecutive syndrome		
		ID		
		SCZ		
3q29	Deletion	ASD	GI abnormalities	[77, 78, 79, 80, 81]
		BPD	Microcephaly	
		DD	Cleft palate	
		SCZ	Chest wall deformity	
			Cardiac malformations	
15q11.2	Deletion	DD	CHD	[82, 83, 84, 85]
		Epilepsy	Ataxia/balance issues	
		NDDs	Facial dysmorphism	
		SCZ		
		SLD		
15q13.3	Deletion	ASD	Mild facial dysmorphism	[8,86]
		Epilepsy	Skeletal defects	
		OCD		
		SCZ		
16p11.2	Deletion	ADHD	High BMI	[30,87]
		ASD	Hypotonia	
		ID	Macrocephaly	
16p11.2	Duplication	ADHD (Dup > Del)	Low BMI	[30,87,88]
		ID	Microcephaly	
		SCZ		
22q11.2	Deletion	Anxiety Disorders	CHD	[32,47,51,58,61,89, 90, 91]
		Early-onset Parkinson’s Disease	Craniofacial abnormalities	
		Psychotic disorder	Hypoparathyroidism	
			Immunodeficiency	
22q11.2	Duplication	ASD	CHD	[26^••^,^92^, ^93^, ^94^]
		Protective against schizophrenia	Craniofacial abnormalities	
			Gastric reflux	

ASD: Autism Spectrum Disorder, ADHD: Attention Deficit Hyperactivity Disorder, BPD: Bipolar Disorder, ID: Intellectual Disability, DD: Developmental Delay, CHD: Congenital Heart Disease, GAD: Generalised Anxiety Disorder, GI: Gastrointestinal, ID: Intellectual Disability, NDDs: Neurodevelopmental Disorders, SCZ: Schizophrenia, SLD: Specific Learning Disorder.

### Clinical evaluation: children

Children with neuropsychiatric CNVs display substantial clinical heterogeneity, which underscores the importance of accessing appropriate multi-specialist care, from speech and language therapists assisting language skills in one carrier with palatal abnormalities and delayed development, to an older child having growth difficulties during puberty. Collateral information from family members can give important insight into prenatal abnormalities or complications, developmental milestones and give an understanding of the patients’ social interactions. Genetic counselling and cascade genetic testing in family members may be appropriate to determine if the variant is *de novo* or inherited and whether other family members are affected.

A thorough history should be obtained from patients and their family, systematically covering medical and psychiatric morbidity. Children with CNVs have a range of neuropsychiatric deficits which cross traditional diagnostic boundaries and comorbidity is common [29^••^]. In managing these, clinicians should carefully consider the following: disparate disorders such as ID, ADHD, schizophrenia and autism have considerable overlap at phenomenological and genetic levels [34, 35, 36]. Moreover, comorbidity can result in diagnostic overshadowing, whereby all clinical symptoms are attributed to a primary diagnosis rather than to additional comorbidity. This occurs particularly in cases of ID [37], and can impede diagnosis of other treatable conditions. CNV carriers are also likely to experience clinically impairing symptoms which do not fit traditional categorical diagnoses, and clinicians should consider dimensional and functional domains of impairment such as attention, social functioning, affect and executive functioning [38] rather than focussing solely on categorical diagnostic criteria. One illustrative example is that many carriers do not meet criteria for autism diagnosis yet have clear functional impairments in domains such as social functioning [39^•^]. Also diagnosing psychiatric conditions within the context of multimorbid physical health problems can provide challenges, for instance cleft palate problems can make it difficult to assess the communication domain of autism [40]. Multi-informant interviews with parent and child are important, particularly for psychotic phenomena when the child may have experiences that the parent is not aware of [41]. It is important that the mental health needs of the child’s parents are not neglected, as compared to the general UK population parents of CNV carriers have are more likely to experience emotional distress [42^•^]; parents of child CNV carriers may need help to access respite care, social support, disability benefits, and counselling.

As well as psychiatric functioning, cognitive function should be gauged in children, formally through an educational assessment and neuropsychology if available. If access to these is limited locally, at the very least, a thorough clinical and educational history should be taken, augmented by bedside assessment of cognitive function as part of the mental state examination. It is advised that clinicians should consider the cognitive phenotype of the child in relation to that of family members who do not have the CNV. Cognitive function in CNV carriers may be modified by the effects of parental IQ [43], and a carrier with average cognitive function in the context of a highly functioning family may experience social and psychological impacts that need to be addressed.

It is also important for clinicians to consider whether treatments for typically developing children are suitable or require modification for CNV carriers for a variety of reasons including developmental delay, sensory impairments, multimorbidity of physical health problems, and sensitivity to adverse effects [44^••^]. For example, CNV carriers who experience cognitive and social difficulties, may find it difficult to access and benefit from therapies such as cognitive behavioural therapy and adaptations to therapies such as shorter sessions, frequent breaks and repetition of content should be considered [45]. There is also evidence that CNV carriers with autism benefit less from social skills training than children with autism but without a CNV [46^•^].

A comprehensive physical examination is vital to cover a range of potential physical comorbidities [47]. After an initial workup, coordinated care allows careful management of the evolving clinical needs of a carrier through their development. For 22q11.2 deletion, consensus guidelines exist which guide multidisciplinary clinics in conducting a thorough post-diagnosis screening [32], recommendations include immunologic evaluation, calcium levels investigation, parathyroid hormone level checks, ophthalmology, audiology, scoliosis examination, palate evaluation, renal ultrasound, and echocardiogram. For other neuropsychiatric CNVs there is emerging evidence of the complexity of their phenotypes (see Table 1), broadly we recommend that a physical examination that covers the major bodily systems including; heart and circulatory function [15,16], neurological examination [22,23], immunologic evaluation, endocrinology check [20,21], musculoskeletal examination, palate evaluation [19], audiology and ophthalmology. Physical assessment should also consider motor function, integrating physical and occupational therapists if necessary, as this is often impaired in carriers [31]. Follow-up screenings across development are necessary as the clinical phenotype changes with age and new medical features may emerge.

### Clinical evaluation: adults

The clinical evaluation of adults with neuropsychiatric CNVs has specific considerations. Although it is likely that carriers with severe congenital and dysmorphic phenotypes will be identified by paediatric services, carriers with more subtle phenotypes may not be referred to genetic testing until adulthood. Adults may present with a personal or familial history of learning difficulties, neurodevelopmental problems, and congenital abnormalities [25,48^••^,^49^]. Although in many instances the CNV will have occurred *de novo*, the presence of a family history of these conditions may well impact on the health and wellbeing of the index case. The utility of genetic testing in adults has been demonstrated in genomic screening of a US healthcare system, where only a minority of adult CNV carriers (41 of 708) had previously received a genetic diagnosis [48^••^]. CNV carriers described relief at a genetic explanation for a ‘lifelong history of learning and behavioural struggles’.

The majority of clinical studies in CNV carriers have focused on children referred to genetics clinics, but a wide range of physical comorbidities in adults are emerging from population studies of CNV carriers such as those in the UK biobank [26^••^,^50^], and it is, therefore, important that adults are screened broadly for physical symptoms using a multidisciplinary approach. Medical phenotypes associated with CNVs in the UK biobank can be searched online (https://kirov.psycm.cf.ac.uk/).

Adult CNV carriers are at risk for major psychiatric disorders including schizophrenia and depression [8,9^•^], and childhood diagnoses can persist into adulthood [51]. Similar to our recommendations for children, management and treatment should be planned in the context of any cognitive or physical comorbidities, and pharmacotherapy should be based on a consideration of any medical comorbidities and sensitivity to adverse effects [44^••^]. For 22q11.2DS a literature is building on psychopharmacotherapy effectiveness and side effects [44^••^], particularly so for the administration of antipsychotics [52, 53, 54], but further research is needed for other CNVs.

The educational, relationship and employment needs of adult CNV carriers should be considered. Compared to the general population, CNV carriers as a group have lower educational achievement, struggle with employment, and have reduced fecundity [55,56^•^,^57^]. However, it should be highlighted that, although on average adult CNV carriers are more likely to experience such difficulties, there is considerable variability and many adults achieve highly in terms of academic and employment success [55]. Thus, it is important to consider carefully the needs of individual patients. Some CNV carriers are highly vulnerable and may require complex social and psychological support. For instance, individuals with 22q11.2DS are at increased risk of financial, emotional and sexual abuse [58]. There is also a need to consider specialist reproductive counselling, which should build on precedents set in 22q11.2DS, where risks of unsafe sex, and prenatal transmission need to be communicated appropriately [58,59], and should be consolidated and monitored across the reproductive age window [32].

For those carriers identified in childhood, effective transition to adult specialty care from paediatric services is crucial. The current reality in many healthcare systems is that support received from paediatric services often stops as soon as the child reaches the service’s age cut-off with little regard to whether the patient and family still need support, and whether care has been effectively transferred to relevant adult services. Early adulthood offers a clinical juncture for psychiatric reassessment and monitoring of known carriers, given this is this the time when late-onset psychopathology in particular psychosis may manifest [60]. Increasing recognition of CNVs and better longitudinal studies are also beginning to give insights into the long-term health of carriers. As the life-expectancy of CNV carriers increases with better care, attention should be paid to the possibility of neurodegenerative impact within these neurodevelopmental syndromes. For 22q11.2DS it has emerged that carriers have increased risk of early-onset Parkinson’s Disease [61].

## Potential for early intervention

Genomic diagnoses are increasingly being made early in development, and provide potential for genomically informed early intervention to ameliorate later risk. This is already occurring for physical health; it has been recommended that all individuals diagnosed with 22q11.2DS have an echocardiogram regardless of presenting phenotype [32] to identify cardiac abnormalities early. There is also clear potential to develop early intervention approaches for psychiatric outcomes in CNV carriers. For instance, several studies have identified early behavioural predictors of psychotic outcomes in 22q11.2DS, [41,62, 63, 64, 65]. Among 16p11.2 deletion carriers, behavioural changes in satiety precede obesity [66], and early motor development may predict autism in 16p11.2 deletion carriers [67]. A barrier to translating this knowledge clinically is that these predictions have been at the level of the group, and clinical risk algorithms that can be applied to individuals are needed. Despite this, clinical practitioners should be alert for identifying early prodromal changes and be willing to refer to appropriate specialties. Clinicians should also ensure both patient and family are informed to the potential psychiatric sequalae as part of genetic counselling support following genetic diagnosis [68].

## Conclusion

We have laid out recommendations for the clinical evaluation and management of individuals with neuropsychiatric risk CNVs based on current knowledge. We are aware that in most instances these are fairly generic, and constitute little more than good clinical practice, including the need to keep abreast of a rapidly developing field, and to consider the possibility of physical, psychiatric, psychological and social manifestations. To make further advances we need systematically to collect and collate more data on the pleiotropic outcomes of CNVs, life course changes, and the specific impact of different interventions. As other articles in this issue demonstrate, progress is being made, and the clinical knowledge base will improve as findings accrue from longitudinal studies of carriers, population studies and clinical case reports [25].

A current challenge to clinical management is the frequent need for multidisciplinary care across several specialties. Healthcare systems are not necessarily designed to support individuals who require coordinated management across multiple medical specialities. Solutions to this will vary from one healthcare system to another; for example, in the UK, General Practitioners are well placed to coordinate referral to other specialities, but many will require education in understanding the specific risks posed by different CNVs. A promising potential model would be to assign the family a health professional who coordinates care across specialities, navigating the family through the complex health problems of their child. Third sector organisations are currently trialling this model, including the UK ‘Same but Different’ organisation’s ‘Rare Navigator’ service (https://www.samebutdifferentcic.org.uk/rare-navigator). From a psychiatric perspective, there is clearly a need to embed an understanding genetics and genomics in residency training programmes [25], and a need for more cross disciplinary interactions with other specialities in particular medical genetics.

It can be argued that, given the complex admixture of developmental, psychiatric and physical outcomes of CNVs, carriers and their families should have access to specialist clinics in which practitioners have the knowledge and experience to coordinate management and to provide treatment, counselling and support. In this regard, some healthcare systems have introduced multidisciplinary clinics focused on individual CNVs such as 22q11.2 Deletion Syndrome (https://www.22qsociety.org/downloads/22q_Clinics_Around_The_World.pdf), others have created rare disease (for example the Centre for Rare Diseases at the University Hospital Birmingham, UK, https://www.uhb.nhs.uk/centre-for-rare-diseases.htm) and behavioural genetic services (for example the Autism Assessment and Behavioural Genetics Service at the Maudsley hospital, UK, https://www.slam.nhs.uk/our-services/service-finder-details?CODE=SU0295) that provide support for a range of genomic conditions. Further, serious consideration is needed by health systems and policymakers so such services can be offered more widely to those with CNVs and other genomic conditions.

In conclusion, individuals with neuropsychiatric CNVs require multi-system assessment and may need co-ordinated, individualised, multidisciplinary care depending on the nature and extent of multimorbidity. Assessment and care should also include consideration of a range of biological, psychological and social factors. Management should be developmentally appropriate, evolve across the life course, and clinicians should be alert for opportunities to instigate early intervention to ameliorate later risk. As CNVs and other genomic conditions are increasingly recognised, health care systems will need to adapt so they can provide appropriate individualised care to their patients.

## Conflict of interest statement

MJO reports a grant from Takeda Pharmaceuticals outside of the submitted work. SJRAC and CJW declare no competing interests.

## References and recommended reading

Papers of particular interest, published within the period of review, have been highlighted as:

• of special interest
•• of outstanding interest

## Appendix A List of useful resources

We have curated a list of resources for clinicians and researchers that provides detailed information on the clinical phenotypes of a range of copy number variants.

### Max Appeal! Consensus document for 22q11.2 Deletion Syndrome

The Consensus Document is a comprehensive but practical and accessible information resource which has had contributions from major centres across the UK, stakeholder organisations, families and over 50 experts (either as authors or advisers) working in the major clinical fields associated with 22q11 deletion. The Committee hopes that the guidance and information supplied will be of significant material benefit to all patients and families and those who provide care and support to them. In particular, given the heterogeneous clinical impact of 22q11DS, it is hoped that the document will be of broad professional interest, relevance and utility.

https://www.maxappeal.org.uk/knowledge/consensus_document

### Simons Searchlight resource for autism associated genetic variants

Simons Searchlight is a partnership of leading scientists, researchers, and families. Simons Searchlight has compiled resources and information on a range of copy number variants and genes related to autism.

https://www.simonssearchlight.org/research/what-we-study/

Unique — The Rare Chromosome Disorder Support Group

Unique is a voluntary organization dedicated to promoting awareness of rare chromosomal abnormalities. The Unique charity has developed free Information Guides to specific chromosome and gene disorders, as well as guides translated into various languages. Guides written by Unique are reviewed on a voluntary basis by clinical geneticists and other professionals working in this field.

https://www.rarechromo.org/disorder-guides/

### Medical phenotypes of CNV carriers in the UK biobank

Data from UK biobank on the medical phenotypes have been made searchable for researchers [26^••^,^50^] (https://kirov.psycm.cf.ac.uk/). It should be noted that data are provided ‘as is’, and without warranty, for scientific and educational use only.

### Measuring the Impact on NeuroDevelopment of CNV (MIND·CNV)

This tool was created to help clinicians estimate the cognitive effect of undocumented deletions based on the results of Huguet *et al*. [95]. The tool estimates the loss of intellectual quotient (IQ) points related to a deletion as well as the estimated probability for that deletion to be *de novo*. (http://www.minds-genes.org/Site_EN/CNVsPredictionTools.html).
